# ProLesA-Net: A multi-channel 3D architecture for prostate MRI lesion segmentation with multi-scale channel and spatial attentions

**DOI:** 10.1016/j.patter.2024.100992

**Published:** 2024-05-15

**Authors:** Dimitrios I. Zaridis, Eugenia Mylona, Nikos Tsiknakis, Nikolaos S. Tachos, George K. Matsopoulos, Kostas Marias, Manolis Tsiknakis, Dimitrios I. Fotiadis

**Affiliations:** 1 Biomedical Research Institute, FORTH, 45110 Ioannina, Greece; 2 Biomedical Engineering Laboratory, School of Electrical & Computer Engineering, National Technical University of Athens, 9 Iroon Polytechniou St., 15780 Athens, Greece; 3 Unit of Medical Technology and Intelligent Information Systems, Department of Materials Science and Engineering, University of Ioannina, 45110 Ioannina, Greece; 4 Institute of Computer Science, FORTH, Heraklion, Greece; 5 Computational Biomedicine Laboratory, FORTH, Heraklion, Greece

**Keywords:** deep learning, magnetic resonance imaging, prostate lesion segmentation, multi-scale attention, cancer detection, medical imaging

## Abstract

Prostate cancer diagnosis and treatment relies on precise MRI lesion segmentation, a challenge notably for small (<15 mm) and intermediate (15–30 mm) lesions. Our study introduces ProLesA-Net, a multi-channel 3D deep-learning architecture with multi-scale squeeze and excitation and attention gate mechanisms. Tested against six models across two datasets, ProLesA-Net significantly outperformed in key metrics: Dice score increased by 2.2%, and Hausdorff distance and average surface distance improved by 0.5 mm, with recall and precision also undergoing enhancements. Specifically, for lesions under 15 mm, our model showed a notable increase in five key metrics. In summary, ProLesA-Net consistently ranked at the top, demonstrating enhanced performance and stability. This advancement addresses crucial challenges in prostate lesion segmentation, enhancing clinical decision making and expediting treatment processes.

## Highlights

• MSSE and MSAG deep-learning attention mechanisms are introduced and assessed separately
• ProLesA-Net model for prostate lesion segmentation is proposed
• The proposed model is compared to six deep-learning models on two datasets
• The comparisons focus on small (<15 mm) and intermediate (15–30 mm) lesions

## The bigger picture

Clinical identification of prostate lesions relies on biparametric MRI analysis. However, several technical limitations prevent the precise segmentation of lesions in the small size range. Deep learning, a type of machine learning consisting of several interconnected layers that process information, for example, MRI images, to generate informative outputs, is a promising strategy in the development of precise treatment strategies to assist MRI analysis and diagnostics. This paper presents a deep-learning framework to segment and identify small- to intermediate-sized prostate lesions. Of course, integrating deep-learning-based tools into clinical practice does not come without challenges, but architectures such as the one introduced in this work demonstrate the potential as well as the limitations of these technologies.

## Teaser

This study presents ProLesA-Net, a deep-learning algorithm tailored for the segmentation of prostate lesions in biparametric MR images, leveraging multi-scale squeeze and excitation and attention gate mechanisms. Evaluated against existing multi-channel 3D segmentation models, ProLesA-Net achieves increased performance in detecting both small and intermediate lesions, thus enhancing the segmentation accuracy and efficiency of clinical diagnoses and treatment planning. Its increased performance, evidenced by testing on diverse datasets, marks an advancement in the application of artificial intelligence for medical imaging.

## Introduction

Prostate cancer (PCa) ranks as the primary most frequently diagnosed malignancy in men, constituting approximately 7.1% of all cancer cases and contributing to 1.4 million new cases every year.^1^ While the 5-year survival rate for early-stage diagnoses is nearly 100%, this rate plunges to as low as 28% for advanced-stage PCa.^2^ Within the diagnostic framework for PCa, accurate delineation of the lesions is of paramount importance, to ensure precise tumor localization and size assessment, early and accurate detection of PCa is indispensable for devising effective therapeutic interventions and enhancing patient prognosis. Among imaging techniques, magnetic resonance imaging (MRI) is predominantly utilized for PCa diagnosis,^3^ due to its superior soft-tissue contrast and multi-parametric imaging capabilities. However, the segmentation of PCa from MRI scans remains a complex endeavor, due to the inherent variability of prostate tissue and subtle distinctions in cancer-affected areas. These complexities are further amplified by differences in imaging protocols, hardware manufacturers, and clinical practices across various healthcare facilities. In addressing these challenges, the incorporation of deep learning (DL) methodologies has shown considerable promise for augmenting the precision and reliability of PCa segmentation. The employment of sophisticated DL techniques enables the automation and standardization of the otherwise labor-intensive manual delineation of lesions, thereby mitigating inter-observer discrepancies and enhancing the consistency of results.

### Related work

According to the literature, several DL approaches have been implemented for prostate lesion segmentation. In a study by Cao et al.,^4^ the authors propose a meta-learning convolutional neural network (CNN)-based approach, whereby they combine the probabilistic outcomes of a CNN feature extractor with conditional random fields and a mutual finding loss function to effectively segment prostate lesions from biparametric MR images acquired from a 3T Siemens scanner. Their dataset included 397 preoperative cases from patients who had undergone post-radical prostatectomy, and the inclusion criteria were that at least one lesion has a Gleason score (GS) >6 or a lesion diameter ≥10 mm, while they obtained a Dice score of approximately 40%. In another study a radiomic-informed U-Net architecture^5^ was proposed to address the task of prostate lesion segmentation in T2-weighted (T2W) MR images acquired by a 1.5 T Philips Ingenia scanner.^6^ The cohort consisted of 2,071 training and validation images from 40 patients, with 415 frames from ten cases reserved for testing, whereby a Dice score of 91.76% was achieved, outperforming U-Net and a couple of DL architectures. Furthermore, Eidex et al.^7^ introduced a region-of-interest (ROI) scoring CNN strategy that (1) is capable of locating the ROI (prostate lesion), (2) assigns scores based on the segmentation of dominant intraprostatic lesions, and (3) segments further the lesion via a cascaded CNN. The authors utilized a cohort of 77 T1-weighted MRI patients originating from Emory Clinic, acquired from 3T Siemens Aera scanner. Their methodology achieved a Dice score of 85% in comparison with Mask R-CNN, U-Net, and nnU-Net. Gunashekar et al.^8^ proposed a Grad-Cam-based U-Net architecture trained on 122 patients to segment prostatic lesions and validated these on 15 co-registered whole-mount histopathology images, acquired from 1.5 T to 3 T Siemens scanners, whereby their methodology led to a Dice score of 31%.

Song et al.^9^ employed a deep multi-scale Attention U-Net for prostate lesion segmentation. They state that their attention mechanism captures the low-resolution features along with the high-resolution ones, leading to adaptive realignment of the context between feature maps, thus providing more informative features, whereby they obtained a Dice score and sensitivity of 70% and 86.5%, respectively. Their cohort included 97 patients from the PROSTATEX challenge,^10^^,^^11^ while the inclusion criteria were the selection of patients with GS ≥ 6. Ren et al.^12^ proposed a 3D encoder/decoder-based network that incorporated densely connected CNN blocks, attention mechanisms, and group normalization atrous spatial pyramid pooling layers whereby they obtained a Dice score of 93.9% in a 4-fold cross-validation scheme and a holdout set 30% from the complete dataset. Their dataset included 180 diffusion-weighted imaging (DWI) MRI cases from 122 healthy individuals and 58 patients with PCa, acquired from a 3 T General Electric scanner. Simeth et al.^13^ investigated the performance of five DL architectures, namely, Unet, Unet++, ResUnet, multiple-resolution residually connected network, and fast panoptic segmentation to segment dominant intraprostatic lesions from a cohort of 365 apparent diffusion coefficient (ADC) patients obtained from 1.5 T General Electric and 3 T Philips and Siemens scanners. They performed the validation on two external private sets, achieving a Dice score of 60% and 45%, respectively for each dataset.

### Purpose of this study

Most of the aforementioned DL models are trained and validated on single-channel inputs, for example,^12^^,^^13^ where the authors utilized only ADC and/or DWI MR sequences, respectively, or the amount of data is limited^6^ and thus the models are less informed regarding the variations induced by various MRI scanners. Furthermore, the unique nature of prostate lesions requires the feature extraction process to be applied on multiple spatial dimensions scale due to extreme lesion size variations.^14^ It is of paramount importance for automated lesion detection models to capture even the smallest of the lesions, as they also pose a threat.^15^

In this regard, the main contributions of this paper are as follows.

(1) To introduce a multi-scale squeeze & excitation attention (MSSE) mechanism alongside a multi-scale attention gate (MSAG) mechanism. These mechanisms are tailored to efficiently propagate lesion-relevant features while simultaneously alleviating the computational burden posed by an extensive number of model parameters. They both expand the notion of squeeze & excitation (SE) and attention gate (AG) mechanisms.
(2) To propose ProLesA-Net, a multi-scale squeeze-and-excite Attention-Net architecture specifically designed for the segmentation of prostate lesions.
(3) To evaluate the performance of ProLesA-Net against six multi-channel 3D DL architectures: 3D U-Net, VNet, Transformer U-Net 3D, USE-Net 3D, nnU-Net, and Attention U-Net 3D. Comparisons are drawn using two separate datasets, comprising a total of 219 and 82 patients, obtained from 3 T Siemens and Philips MR scanners from multiple clinical sites.
(4) To assess the models’ efficacy specifically on smaller and intermediate lesions with axial diameters less than 15 mm and between 15 and 30 mm, respectively.

## Results

Table 1 reports the performance metrics for each model evaluated using the Prostate-158 external validation dataset. ProLesA-Net outperforms the competing models in five out of five key metrics. Specifically, in terms of Dice score, ProLesA-Net records a score of 30.56%, outclassing the runner-up model, Attention U-Net, which scores 28.24%. Furthermore, the ProLesA-Net achieves a recall rate of 33.91%, slightly surpassing Attention U-Net’s 33.67%, thereby demonstrating its capability in accurately identifying lesion voxels that are consistent with the ground truth. In the category of distance-based metrics, the ProLesA-Net achieves the best performance, with 19.61 mm Hausdorff distance compared to nnU-Net’s 20.06 mm. This suggests that ProLesA-Net yields on average fewer worst-case distances between the ground truth and the PR. Similarly, ProLesA-Net leads the field in average surface distance with a measurement 4.43 mm, underscoring its superiority in generating predictions in better agreement with the ground truth, while the second best, nnU-Net, achieved 4.98 mm.

**Table 1:** Mean values for Dice score, recall, and precision and median values for Hausdorff distance and average surface distance in the external validation dataset (Prostate 158)

Model	Dice score (%)	Hausdorff distance (mm)	Average surface distance (mm)	Recall (%)	Precision (%)	Parameters (millions)
U-Net	27.38	24.60	5.11	31.69	33.66	18,335,585
VNet	24.03	26.66	5.93	25.16	31.61	18,641,089
TransU-Net	21.79	21.77	5.99	22.10	30.85	41,392,417
USE-Net	26.39	24.09	5.23	28.39	32.23	18,711,649
nnU-Net	24.18	20.06	4.98	20.68	36.17^a^	–
Attention U-Net	28.24	24.83	5.35	33.67	32.19	16,302,309^a^
ProLesA-Net	30.56^a^	19.61^a^	4.43^a^	33.91^a^	33.72	16,544,129

a Best score

However, in PCa, lesions with a diameter spanning between 0 and 15 mm are considered the most challenging for any model to detect.^8^ To evaluate the performance of our model in such cases, we carried out a subgroup analysis including only small lesions (axial diameter <15 mm), which make up 61.5% of the total cases. Table 2 provides the results of all baseline methods and the proposed ProLesA-Net model, which exhibits the highest Dice score, 22.33%, outperforming by a large margin other models such as the U-Net (18.17%) and Attention U-Net (18.99%). The U-Net model has the lowest average surface distance at 6.69 mm, marginally outperforming the 6.74 mm of the ProLesA-Net model. This minor difference in average surface distance is outweighed by the substantial improvement in Hausdorff distance achieved by the ProLesA-Net (21.98 mm vs. U-Net’s 27.74 mm). In terms of recall, the ProLesA-Net again outperforms all other models with a score of 27.54%, while it scores third in terms of precision (24.21%).

**Table 2:** Mean values for Dice score, recall, and precision and median values for Hausdorff distance and average surface distance for lesions with axial diameter <15 mm on the external validation dataset (Prostate 158)

Axial lesion diameter (mm)	Model	Dice score (%)	Hausdorff distance (mm)	Average surface distance (mm)	Recall (%)	Precision (%)
0–15	U-Net	18.17	27.74	6.69^a^	22.30	25.17
	VNet	15.00	27.03	8.31	17.68	20.27
	TransU-Net	14.03	22.07	9.15	15.60	19.85
	USE-Net	18.01	24.51	8.16	21.50	20.58
	nnU-Net	14.40	26.21	9.65	12.17	25.30^a^
	Attention U-Net	18.99	30.30	8.03	25.14	21.81
	ProLesA-Net	22.33^a^	21.98^a^	6.74	27.54^a^	24.21

a Best score

Since we evaluated our model’s performance on the small-lesion subgroup, it is also of interest to evaluate its performance on the subset with lesions exhibiting medium axial diameters (15–30 mm), which constitute 34% of the total cases. A detailed presentation of these results can be found in Table 3. In general, the proposed model, ProLesA-Net, outperforms the baseline methods in three out of five segmentation metrics, achieving 39.63% Dice score, 14.43 mm Hausdorff distance, and 3.10 mm average surface distance. The second best method for each metric varies among Attention U-Net, nnU-Net, and U-Net. When considering recall, the ProLesA-Net comes second with a score of 41.55%, while Attention U-Net comes first at 42.53%. Regarding precision, nnU-net surpasses all the models, reaching 47.13%, while the other models had comparable performance ranging from 41% to 45%.

**Table 3:** Mean values for Dice score, recall, and precision and median values for Hausdorff distance and average surface distance on lesions with axial diameter between 15 and 30 mm on the external validation dataset (Prostate 158)

Axial lesion diameter (mm)	Model	Dice score (%)	Hausdorff distance (mm)	Average surface distance (mm)	Recall (%)	Precision (%)
15–30	U-Net	35.80	22.41	3.55	41.08	41.99
	VNet	32.80	26.65	4.61	32.82	43.74
	TransU-Net	28.15	26.38	4.72	27.72	42.34
	USE-Net	33.83	25.02	3.81	34.48	45.33
	nnU-Net	33.97	15.61	3.94	29.29	47.13^a^
	Attention U-Net	37.28	22.27	4.18	42.53^a^	43.74
	ProLesA-Net	39.63^a^	14.43^a^	3.10^a^	41.55	43.68

a Best score

**Figure 1:**
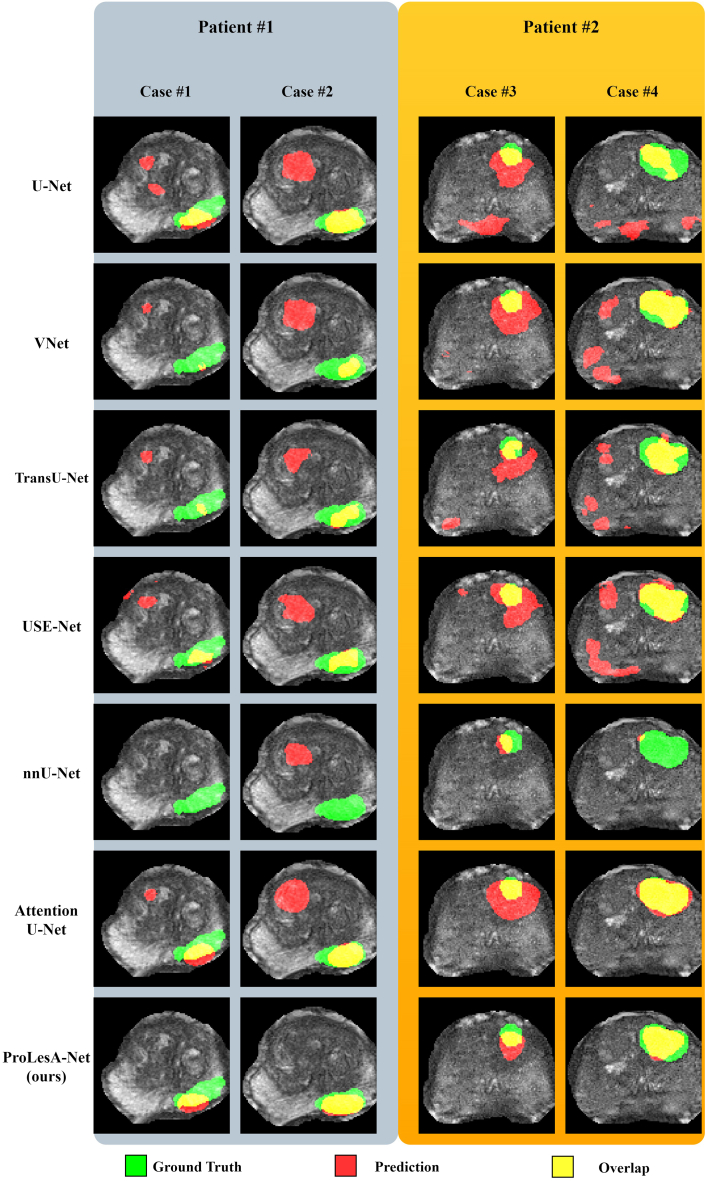
The predictions of each model and the ground-truth segmentations for two patients shown in two successive axial slices

In Figure 1, the performance of various DL models for prostate lesion segmentation on the T2W MR sequences of two patients is presented. For each patient, we have delineated two distinct cases, which correspond to successive T2W images captured along the axial plane. The strategic presentation of these cases in the figure is designed to illuminate not solely the intraplanar heterogeneity of predictions but also to underscore the variation that occurs across sequential axial slices. This dual emphasis allows for a more comprehensive evaluation of the models’ performance in terms of spatial consistency and the integrity of 3D lesion characterization. The predictions reveal a common trend among the majority of the models to erroneously predict multiple clusters of false positives. This is particularly evident in the spatial distribution of the segmentations, where many architectures, such as TransU-Net, are observed to produce as many false-positive regions as the ground-truth regions.

Diverging from this pattern, ProLesA-Net demonstrates a significant reduction in false positives. However, it is in the consistency across the axial slices where ProLesA-Net distinguishes itself. Unlike other models, ProLesA-Net maintains its efficacy when evaluating continuity across successive axial slices. This is exemplified in patient #1, cases #1 and #2 where ProLesA-Net exhibits fewer false positives relative to other models, particularly over continuous slices, thereby encapsulating a more coherent and clinically relevant 3D lesion structure.

Overall, our model is consistently achieving the best performance in the three major segmentation-related metrics (i.e., Dice score, Hausdorff distance, and average surface distance), indicating its robustness and generalizability. Furthermore, it stands out as the sole model exhibiting consistency across lesion sizes, proving to be the most effective by minimizing the generation of false-positive areas compared to other tested models. At the same time, its low complexity (comparatively low number of parameters) makes it highly efficient and suitable for deployment in resource-constrained environments, ensuring faster processing times without compromising its accuracy.

### Ablation experiments

#### Comparisons with baseline and attention mechanisms

In Table 4, the ablation studies regarding the impact of each attention mechanism, AG, SE, MSAG, MSSE, and their combinations in the baseline model are demonstrated with respect to lesion axial diameters. The baseline model consists of typical convolution layers as showcased in Figure 1, without having any multi-scale attention mechanism. Notably, the combination of baseline + MSAG + MSSE yielded the highest Dice score, indicating increased segmentation accuracy, particularly with an improvement of up to 30.56% for all lesions and 39.63% for lesions in the 15- to 30-mm diameter range. This model also demonstrated the lowest Hausdorff distance at 19.61 mm for all lesions and 14.43 mm for lesions between 15 and 30 mm in diameter, suggesting a closer match to the ground-truth boundary. Additionally, the average surface distance was markedly reduced to 4.43 mm for all lesions and further to 3.10 mm for lesions of 15–30 mm, underscoring the model’s precision in estimating lesion surfaces. The precision metric further supports the increase in performance of the baseline + MSAG + MSSE model, with precision peaking at 33.72% for all lesions and 43.68% for lesions of 15–30 mm, indicating a high rate of true-positive detections relative to the total number of positives. Comparing baseline + MSAG with baseline + AG, the results indicate the benefits of the MSAG mechanism. For all lesions, baseline + MSAG shows a higher Dice score of 25.57% compared to baseline + AG’s 25.03%, demonstrating a slight but noticeable improvement in segmentation accuracy. More pronounced is the reduction in the Hausdorff distance from 28.35 mm with AG to 21.15 mm with MSAG, indicating a more accurate approximation of the lesion boundaries. The average surface distance also sustains a significant decrease from 7.20 mm to 5.41 mm. These improvements carry through the recall and precision metrics as well, with baseline + MSAG achieving higher precision (25.74%) compared to baseline + AG (22.84%), which suggests more accurate lesion detection.

**Table 4:** Mean values for Dice score, recall, and precision and median values for Hausdorff distance and average surface distance for the whole external testing dataset (Prostate 158), and for baseline with AG, baseline with MSAG, baseline with SE, baseline with MSSE, baseline with AG and SE, and baseline with MSAG and MSSE

Axial lesion diameter (mm)	Model	Dice score (%)	Hausdorff distance (mm)	Average surface distance (mm)	Recall (%)	Precision (%)
All lesions	baseline + AG	25.03	28.35	7.20	46.21^a^	22.84
	baseline + MSAG	25.57	21.15	5.41	35.72	25.74
	baseline +SE	20.40	32.10	10.27	38.38	17.79
	baseline + MSSE	22.52	29.42	8.43	42.40	22.09
	baseline + AG + SE	24.15	23.29	5.72	38.46	27.80
	baseline + MSAG + MSSE (ProLesA-Net)	30.56^a^	19.61^a^	4.43^a^	33.91	33.72^a^
0–15	baseline + AG	13.41	31.31	11.94	38.39^a^	13.68
	baseline + MSAG	13.75	24.13	11.82	26.65	14.13
	baseline +SE	10.08	36.01	14.47	35.46	8.53
	baseline + MSSE	12.15	33.05	11.99	33.36	12.99
	baseline + AG + SE	12.41	28.76	11.91	26.76	20.77
	baseline + MSAG + MSSE (ProLesA-Net)	22.33^a^	21.98^a^	6.74^a^	27.54	24.21^a^
15–30	baseline + AG	33.25	24.90	5.00	54.02^a^	32.00
	baseline + MSAG	34.94	19.68	4.31	44.80	37.36
	baseline +SE	27.71	29.87	8.14	43.40	27.05
	baseline + MSSE	29.86	28.09	6.08	49.34	31.19
	baseline + AG + SE	32.46	21.88	4.78	50.16	34.83
	baseline + MSAG + MSSE (ProLesA-Net)	39.63^a^	14.43^a^	3.10^a^	41.55	43.68^a^

a Best score

When comparing baseline + MSSE to baseline +SE, the introduction of the MSSE mechanism also demonstrates enhanced model performance. The Dice score increases from 20.40% with SE to 22.52% with MSSE for all lesions, indicating better overall segmentation accuracy. The Hausdorff distance shows a decrease from 32.10 mm with SE to 29.42 mm with MSSE, and the average surface distance improves from 10.27 mm to 8.43 mm, both of which underscore the enhanced boundary delineation and surface approximation capabilities of the MSSE mechanism. The recall and precision metrics further reflect this improvement, with baseline + MSSE showing a better balance between the detection of true positives and the avoidance of false positives.

#### Comparisons with U-Net and multi-scale attention mechanisms

In Table 5 the results for U-Net and each multi-scale attention mechanism are presented. The integration of MSSE and MSAG mechanisms into the U-Net model has resulted in evident improvements in the lesion segmentation performance over a range of lesion sizes. More specifically, U-Net + MSAG + MSSE achieved a Dice score of 29.75%, indicating significant improvement in comparison to the U-Net model. The model’s stronger ability for accurately identifying lesion boundaries is highlighted by an improvement in Hausdorff distance to 20.51 mm and an average surface distance of 4.53 mm. The U-Net + MSAG + MSSE combination demonstrates significant proficiency in analyzing small lesions (0–15 mm) as well, effectively overcoming the inherent obstacles associated with their small size and ambiguous characteristics. This configuration distinguishes itself from others with a Dice score of 21.91%. Additionally, it achieves a decrease in Hausdorff distance to 22.64 mm and maintains an average surface distance of 6.91 mm. The model exhibits strong and efficient performance in segmenting lesions of intermediate size (15–30 mm), obtaining a Dice score of 39.49%. The performance of this configuration is comparable to that of the U-Net + MSAG, but it demonstrates noticeable enhancement by improving the Hausdorff distance to 16.76 mm and reaching the dominant average surface distance of 3.14 mm among the examined configurations.

**Table 5:** Mean values for Dice score, recall, and precision and median values for Hausdorff distance and average surface distance for the whole external testing dataset (Prostate 158) and for U-Net with MSAG, U-Net with MSSE, and U-Net with MSAG and MSSE

Axial lesion diameter (mm)	Model	Dice score (%)	Hausdorff distance (mm)	Average surface distance (mm)	Recall (%)	Precision (%)
All lesions	U-Net	27.38	24.60	5.11	31.69	33.66^a^
	U-Net + MSAG	27.64	20.58	4.93	33.88	31.62
	U-Net + MSSE	27.17	22.53	4.87	35.35	31.76
	U-Net + MSAG + MSSE	29.75^a^	20.51^a^	4.53^a^	37.79^a^	31.87
0–15	U-Net	18.17	27.74	6.69^a^	22.30	25.17^a^
	U-Net + MSAG	18.37	24.23	7.35	25.62	20.09
	U-Net + MSSE	18.82	23.00	7.54	29.45	22.96
	U-Net + MSAG + MSSE	21.91^a^	22.64^a^	6.91	34.69^a^	22.78
15–30	U-Net	35.80	22.41	3.55	41.08	41.99
	U-Net + MSAG	41.40	17.76	3.52	46.15^a^	47.55^a^
	U-Net + MSSE	39.57^a^	18.37	3.76	44.13	44.83
	U-Net + MSAG + MSSE	39.49	16.76^a^	3.14^a^	42.39	45.37

a Best score

Table 6 showcases the comparison between ProLesA-Net and multi-scale attention enhanced U-Net. Overall, as evidenced by Tables 1, 2, 3, and 4 and Figure 1, the main characteristic of the MSSE and MSAG mechanisms is the significant reduction in Hausdorff distance on both backbone models. However, ProLesA-Net demonstrates better performance in the majority of evaluation metrics, while it contains fewer parameters than U-Net + MSAG + MSSE. In our work the backbone component of ProLesA-Net contains fewer convolutional operations than U-Net’s architecture, especially in the encoder and decoder parts where we perform a single convolution operation rather than two as in the case of U-Net. Furthermore, we were inspired by the backbone architecture of the nnU-Net^16^ model and its automatic configuration it performs before the training procedure. That said, ProLesA-Net showcases an improvement especially in Hausdorff distance by 0.9 mm, 0.66 mm, and 2.33 mm for all lesions, 0–15 mm lesions, and 15–30 mm lesions, respectively. Further, for average surface distance the trend is similar, whereas ProLesA-Net improves this metric for 0.1 mm, 0.17 mm, and 0.04 mm for all lesions, 0–15 mm lesions, and 15–30 mm lesions, respectively.

**Table 6:** Comparison between ProLesA-Net and U-Net + MSAG + MSSE for the whole external testing dataset (Prostate 158)

Axial lesion diameter (mm)	Model	Dice score (%)	Hausdorff distance (mm)	Average surface distance (mm)	Recall (%)	Precision (%)
All lesions	U-Net + MSAG + MSSE	29.75	20.51	4.53	37.79^a^	31.87
	ProLesA-Net	30.56^a^	19.61^a^	4.43^a^	33.91	33.72^a^
0–15	U-Net + MSAG + MSSE	21.91	22.64	6.91	34.69	22.78
	ProLesA-Net	22.33^a^	21.98^a^	6.74^a^	27.54^a^	24.21^a^
15–30	U-Net + MSAG + MSSE	39.49	16.76	3.14	42.39	45.37^a^
	ProLesA-Net	39.63^a^	14.43^a^	3.10^a^	41.55^a^	43.68

a Best score

## Discussion

The primary objective of the present study is to introduce ProLesA-Net, a DL architecture explicitly designed for the segmentation of prostate lesions using biparametric MRI. The novelty of ProLesA-Net lies in the integration of MSSE and MSAG into its architecture. These mechanisms serve to streamline the propagation of features while selectively amplifying the contribution of lesion-relevant voxels across varying spatial resolutions. Our experiments reveal that ProLesA-Net outperforms competing models across multiple evaluation metrics when assessed on an external validation dataset, comprising 82 patient cases and approximately 5,904 images. Importantly, the proposed architecture demonstrates remarkable effectiveness in segmenting challenging lesions with small axial diameters (less than 15 mm), while it was also superior regarding the identification of intermediate lesions (15–30 mm). This consistent performance across different lesion sizes underscores the model’s adaptability and precision.

As shown in Figure 1, a prominent challenge observed across the segmented slices is the escalation in the frequency of false-positive predictions generated by various models. However, the integration of multi-scale double attention mechanisms (MSSE and MSAG) appears to mitigate this issue by enabling the architecture to generate predictions with reduced susceptibility to false-positive voxels. The enhanced representational capabilities conferred by these attention mechanisms have been elaborated upon in Zaridis et al.^27^ Despite incorporating attention mechanisms, Attention U-Net and USE-Net do not effectively address the problem of false positives due to the absence of multi-scale connections and multi-dimensional attentions, whether spatial or channel-wise. Conventional CNN architectures and transformer-based models yielded a high rate of false positives, rendering them in clinical practice where they may impact radiologists’ diagnosis.^17^ These findings underscore the necessity for specialized architectural designs tailored to tackle complex tasks, such as the segmentation of prostate MRI lesions.

In prior studies, numerous investigations have been undertaken to address the segmentation of prostate MRI lesions. A critical limitation in these studies is the absence of diverse data sourced from multiple clinical centers, along with the unavailability of biparametric MR sequences for the artificial intelligence (AI) systems, hindering the development of robust and generalizable solutions. For example, Cao et al.^4^ employed a CNN-based framework to segment prostate lesions, though excluding lesions smaller than 10 mm in diameter and those considered clinically insignificant (with a GS of <6). Despite these limitations, they reported a Dice score of 40%. This result is comparable to that achieved by our ProLesA-Net for intermediate lesions, where we also observed a Dice score of approximately 40%. Additionally, Hambarde et al.^6^ evaluated their radiomics-informed U-Net on a dataset consisting of 50 patients (40 for training and 10 for testing), focusing solely on T2W MR sequences. Song et al.^9^ achieved a Dice score of 70% using a single dataset, the Prostate X,^10^^,^^11^ consisting of 97 patients, and evaluated their methodology under a 5-fold cross-validation scheme. However, the urgency for external validation remains unaddressed although it is critical for assessing the generalizability of AI systems across different patient populations and clinical settings.

The robustness of ProLesA-Net is demonstrated through its successful external validation on a highly diverse dataset, encompassing lesions of varying sizes and cancer grades. Furthermore, our model showcases its ability to segment effectively both small lesions (<15 mm), which are considered the most challenging ones, and intermediate lesions (>15 mm). The differentiation of lesions below and above 15 mm has been discussed by An et al.,^18^ who found that a high likelihood of clinically significant PCa is directly related with the 15-mm lesion size threshold. Especially for the small lesions, attention mechanisms, either spatial (Attention U-Net) or channel (USE-Net), delineate their representational power of lesion-related feature extraction by being the second- and fourth-best models, respectively, whereas they also have a reduced false-positive rate. Significant variability in lesion sizes makes the handling of features across different resolutions critical for effectively capturing lesion-specific features. The influence of multi-scale feature fusion in this context has also been examined by Xu et al.^19^ In our study, however, we introduce a methodology that processes features of varying scales within the attention modules and propagates them while reducing dimensions, thereby decreasing the overall size of the model yet preserving high-resolution information. This approach facilitates reducing the number of trainable parameters by limiting the convolutional operations in the expansion pathway.

The distribution of lesion sizes across datasets consists also a topic of discussion. It is of paramount importance for an AI system to be able to transfer its knowledge in external datasets with a diverse distribution of lesion size. In our analysis the training dataset, the one obtained from the PICAI,^20^ consists of 40% of cases with small lesions and 37% of cases with intermediate lesions. However, the dataset used for external validation, Prostate 158,^21^ has a different distribution for small lesions (61.5% of total cases) and a slightly different one for intermediate lesions (34% of total cases). The current scheme implies that a DL model is more prone to be trained on intermediate-related features rather than equally balanced ones. To that extent, ProLesA-Net showcased its transferability of detecting small lesions more efficiently even if the training dataset’s distribution is different. All the tested models highlighted their inability not to overfit on the underlying lesion size distribution of the training data.^19^ The overfitting effect is most dominant on the transformer-based U-Net, where even regularization and dropouts were used, although the performance of transformer-based networks has shown an increased performance for lesion characterization^23^^,^^24^ and in nnU-Net.

### Limitations of the study

Our work has some limitations that should be acknowledged. Initially the reduced number of relatively large lesions on the external validated set, consisting of 4.5% (5 cases) of the total cases, led us to exclude the subgroup analysis for larger lesions (>30 mm). Another external validation dataset with more diverse data regarding lesion size would be beneficial for the inclusion of that category also. Furthermore, the utilization of uncertainty estimation techniques such as Bayesian CNNs^25^ may be used in order to increase the interpretability of DL algorithms more effectively. Moreover, the presence of a lesion within the prostate does not imply that other lesions would not be present. Therefore, a dataset containing multiple prostate MRI lesions would also be beneficial for multiple lesion detection simultaneously. In addition, a variety of image-processing techniques may be utilized for prostate MRI tasks^26^^,^^27^ to enhance the features of the prostate gland further.

### Conclusions

The present study introduces ProLesA-Net, a 3D multi-channel DL architecture tailored explicitly for the segmentation of prostate lesions on biparametric MRI scans. The architecture integrates MSSE and MSAG mechanisms to enhance feature propagation and lesion-relevant voxel attention across diverse spatial scales while retaining a reduced number of trainable parameters. External validation demonstrated that the ProLesA-Net surpasses competing models, including the state-of-the-art nnU-Net. Furthermore, it demonstrated increased efficacy in segmenting small lesions with axial diameter less than 15 mm as well as in identifying lesions of intermediate size.

## Experimental procedures

### Resource availability

#### Lead contact

Prof. Dimitrios I. Fotiadis, e-mail: fotiadis@uoi.gr.

#### Materials availability

The study did not generate new unique reagents.

#### Data and code availability

This paper analyzes existing, publicly available data. The data used for this analysis, which are open access, were obtained from the PICAI challenge ^20^^,^^29^ and the Prostate-158 challenge.^21^^,^^22^

All original code regarding ProLesA-Net along with the MSSE and the multi-scale gate attention mechanisms has been deposited at this GitHub Repository link in python,^28^ implemented in tensorflow 2.7. Also, a demo to assist the reproduction of the current study has been added in the repository along with both of the attention mechanisms for anyone to use separately. The code is also under the following DOI: https://doi.org/10.5281/zenodo.10907658.

### Methods

#### Datasets

In our analysis, two openly available datasets were utilized for training and external validation, namely the PICAI^20^^,^^29^ and the Prostate 158.^21^ The former consists of 219 cases and 15,768 images used for training and validation, and the latter from 82 cases consisting of 5,904 images purposed as external testing. The PICAI dataset contains multi-sequence data acquired by 3T Siemens and 3T Philips scanners from multiple clinical centers, while the Prostate 158 dataset contains data acquired by a 3T Siemens scanner. Therefore, T2W, ADC, and DWI sequences were utilized for the analysis.

#### Preprocessing

For each patient, an affine alignment protocol was applied to co-register ADC and DWI scans with their corresponding T2W images. This choice of T2W as the reference standard was informed by its use for the original delineation of ground-truth lesion masks. As the subsequent analysis focuses only on the prostate’s whole gland (WG), the original WG masks were used to filter out task-agnostic regions. Following this, the acquired volumes were resampled to achieve a uniform pixel spacing and slice thickness 0.5 mm × 0.5 mm × 3.0 mm, guided by the mean spatial dimensions observed across the patient cohort. To ensure compatibility with the requirements of the DL algorithms, a cropping and padding strategy was then employed, yielding the final volume dimensions 24 slices × 192 pixels × 192 pixels. When cropping was deemed necessary, this was based on the guidelines set by Aldoj et al.,^30^ which typically involves the exclusion of the upper and lower quartiles of the initial volume. Finally, the normalization method chosen for this study was the minimum-maximum strategy, which rescaled the voxel values for each imaging sequence to a standardized range from 0 to 1,^31^ thereby aiding in more efficient model convergence.

### Background

#### Deep-learning models

Six DL segmentation architectures were implemented: the 3D U-Net,^32^ the Attention U-Net 3D,^33^ the USE-Net 3D,^34^ The VNet,^35^ the Transformer U-Net 3D,^36^ and the nnU-Net.^16^

The 3D U-Net represents an extension of the original U-Net CNN, tailored specifically for semantic segmentation tasks involving 3D volumetric images. This architecture is fundamentally composed of two primary components, (1) a contraction phase and (2) an expansion phase. The contraction phase engages in downsampling of the input image to capture salient features, while the expansion phase contributes to upsampling these acquired features. During the expansion, the upsampled features are fused with features originally extracted during the contraction phase, culminating in the production of a segmented mask. An augmented version of the 3D U-Net is the Attention U-Net 3D, which consists of AG mechanisms at the beginning of the decoder’s layers. These mechanisms facilitate the entire model to focus on specific regions of the image that are more relevant, especially for lesion segmentation tasks.^37^ Furthermore, the 3D USE-Net serves as an additional encoder-decoder structure, which incorporates SE attention modules^38^ at the tail of each encoding stage. By enabling dynamic recalibration of channel-wise features, the SE attention mechanisms enhance the original 3D U-Net model’s ability to represent lesion structures and shapes. Specifically, the SE blocks are designed to adaptively modulate the significance attributed to individual channels within the feature maps, thereby directing the network’s focus toward the most salient features. Moreover, the VNet architecture^35^ comprises both encoding and decoding pathways, mirroring the structure of the U-Net model. Distinguishing itself, however, VNet integrates residual connections as a supplementary element.

On the contrary, the TransU-Net 3D model integrates the Vision Transformer (ViTs)^39^ architecture as a bottleneck mechanism, situated at the final stage of its contracting pathway. This approach aims to process low-resolution feature maps by treating them as segmented patches and utilizing self-attention algorithms to identify both localized and distant dependencies. Notably, the ViTs’ self-attention scheme diverges from traditional CNNs in a significant manner: while CNNs primarily capture local features via constrained receptive fields, the self-attention modules within ViTs are designed to discern global contextual information across the entire image. Regarded as the current gold standard in medical image segmentation, the nnU-Net distinguishes itself through an automated approach to hyperparameter tuning, enabling robust performance over an extensive array of both 2D and 3D segmentation challenges. Comprising three distinct architectures—3D U-Net, Cascaded U-Net, and 2D U-Net—the nnU-Net offers considerable flexibility. For the scope of our work, we opted to employ the 3D full-resolution U-Net variant.

#### Squeeze and excitation and attention gate mechanisms

The SE layer is an attention-based DL module that emphasizes the recalibration of channel-wise features by representing the interconnections between feature maps. Global spatial information is compressed into a single value using a squeeze operation. This is followed by an excitation operation that utilizes a gating mechanism with a sigmoid activation to capture relationships across feature maps. This procedure enables the network to improve informative characteristics and suppresses fewer irrelevant ones within an individual scale, resulting in enhanced model performance and interpretability.

The AG aims to enhance the sensitivity and specificity of the model by focusing only on relevant spatial regions, hence eliminating the requirement for additional supervision. AG eliminates unnecessary information by producing attention coefficients, which are later utilized to adjust the features related to the area of interest. The utilization of a focused attention mechanism allows the model to concentrate on significant features, resulting in enhanced feature representation.

#### Multi-scale squeeze-and-excite Attention-Net: ProLesA-Net

In this study, the aim is to expand the scope of the original SE layer by including features from different scales before recalibration, in addition to their ability to recalibrate features within a single scale. The objective of this effort is to enhance comprehension of complex information, noting that the integration of several scales is crucial for analysis that requires a comprehensive exploration of both low-resolution and high-resolution characteristics. In the same way, for the MSAG, our approach involves the incorporation of multi-scale characteristics, which are derived from both the MSSE and the expanding path of the network, thereby building upon the principles underlying the original AG layer. Softmax activation function is employed, in contrast to the original network, to allocate attention across the feature maps in the spatial dimensions.

**Figure 2:**
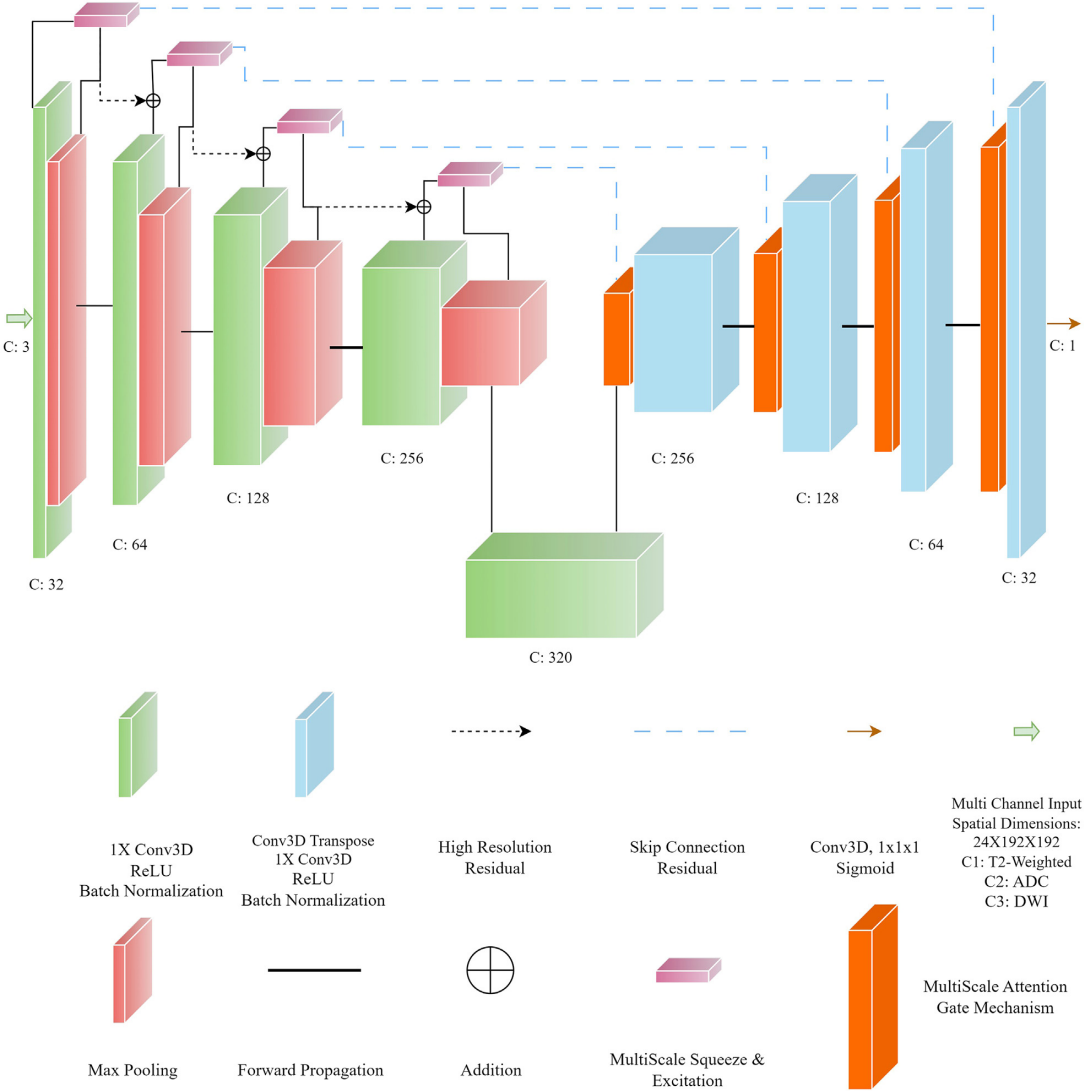
The proposed architecture of ProLesA-Net

Figure 2 illustrates the comprehensive architecture of ProLesA-Net, which comprises an encoder containing convolutional neural networks in each layer, followed by max-pooling operations for spatial dimensionality reduction of the feature maps. A notable limitation of this spatial contraction is the potential loss of critical lesion-specific attributes due to the reduced resolution following the pooling steps. To mitigate this, our MSSE mechanism enables the retention and integration of high-resolution features extracted from earlier layers with those from subsequent lower-resolution layers. These features form the residuals that are subsequently processed through the MSAG at each stage of the decoder. This sequential process amplifies salient features originating from both the encoder and the multi-scale residuals. Additionally, the incorporation of attention mechanisms allows for model size optimization by eliminating specific convolutional operations in the decoder following the implementation of the MSAG.

**Figure 3:**
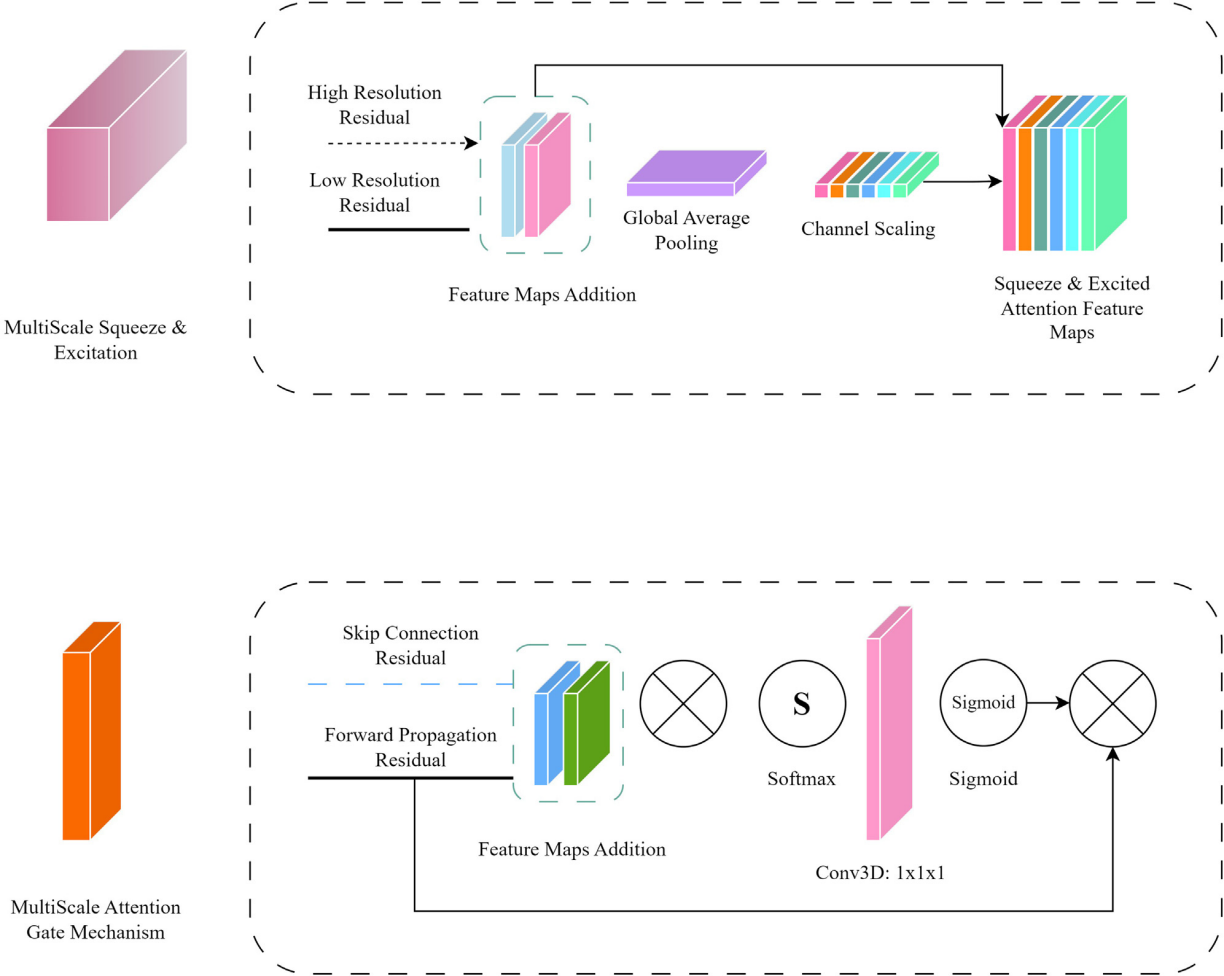
The MSSE mechanism (top) and the MSAG mechanism (bottom)

#### Multi-scale squeeze and excitation and attention gate mechanisms

Figure 3 presents the MSSE and the MSAG mechanisms. For the MSSE mechanism, the high-resolution residuals propagate through a three-dimensional identity convolution layer to produce the same number of feature maps as the low-resolution residuals. This operation is described as

(Equation 1) $${HRR}_{d,w,h}={hrr}_{d,w,h}*I\text{,}$$

where ${HRR}_{d,w,h}$ are the high-resolution residuals after the identity convolutional operation, (d,w,h) are the spatial dimensions as indices for the application position of the kernel, ${hrr}_{d,w,h}$ are the pre-convolved high-resolution residuals, I is the identity matrix, containing values of 1 in its main diagonal, and ∗ is the element-wise multiplication. The low-resolution residuals are downsampled with an identity convolution operation, with a stride of 2 and kernel sizes of 1. The rationale of choosing an identity convolution came from previous works that studied the downsampling effect utilizing convolutional layers when utilized as part of a DL model.^40^ This process serves as an upsampling operation on the low-resolution features maps to match the shape of the high-resolution ones. The high-resolution residuals after the identity convolutional operation, and the low-resolution residuals, are then added in an element-wise manner, given by Equation 2:

(Equation 2) $${FMA}_{d,w,h}=\sum_{dep=0}^{dep=D}\sum_{wd=0}^{wd=W}\sum_{hg=0}^{gh=H}\left({HRR}_{dep,wd,hg}+{lrr}_{dep,wd,hg}\right)\text{,}$$

where ${FMA}_{d,w,h}$ are the feature maps after the aggregation of high-resolution residuals ${HRR}_{dep,wd,hg}$ and low-resolution residuals ${lrr}_{dep,wd,hg}$, while (*D*, *W*, *H*) are the depth, width, and height of the feature map. The feature aggregation process was selected over concatenation to alleviate the computational burden imposed by the latter. For instance, aggregation does not alter the size of the feature maps in the channel dimension, while concatenation merges the features from high-resolution residuals and low-resolution residuals in the channel dimension. Furthermore, previous research has shown that element-wise aggregation can be as effective, if not more so, than concatenation or multiplication. For instance, the success of residual connections in deep networks, which utilize element-wise aggregation to combine features across layers, underscores the effectiveness of this approach in enhancing learning and facilitating deeper architectures without degradation.^41^ Sequentially the aggregated feature maps are pooled globally with the global average pooling operation:

(Equation 3) $$\text{GAP}=\frac{1}{DWH}\sum_{dep=0}^{dep=D}\sum_{wd=0}^{wd=W}\sum_{hg=0}^{gh=H}{FMA}_{dep,wd,hg}\text{,}$$

where GAP is the global average pooled features in each spatial dimension *D*, *W*, *H*. The GAP features are then propagated through two fully connected layers, whereas the first one contains a number of neurons equal to $\frac{1}{8}$ of the number of GAP feature maps and the second one contains an equal number of neurons with the number of GAP feature maps, all followed by a ReLU activation function.^42^ Eventually, an attention score is given in those GAP feature maps and those are multiplied with the initial aggregated feature maps (*FMA*), forming the MSSE feature maps which are going to forward passed at the decoder layer and at the next multi-scale squeeze & excited layer. The outcome is obtained as:

(Equation 4) $$SEout={FMA}_{dep,wd,hg}*\text{GAP}\;\text{.}$$

Regarding the MSAG mechanism, the skip connection residual from each encoder layer (SEout) and the forward propagated residual (FPR), which are the feature maps propagated from the expanding path of the model, are convolved with identity convolutional operations to homogenize them and add them, leading to the feature map aggregation:

(Equation 5) $${FMA}_{d,w,h}=\sum_{dep=0}^{dep=D}\sum_{wd=0}^{wd=W}\sum_{hg=0}^{gh=H}\left({SEout}_{dep,wd,hg}+{FPR}_{dep,wd,hg}\right)*I\text{,}$$

where SEout is the skip connection residual from each encoder layer’s MSSE mechanism and ${FPR}_{d,w,h}$ are the forward propagated residuals. Post aggregation, ${FMA}_{d,w,h}$ is multiplied with the ${FPR}_{d,w,h}$, and the Softmax activation function is applied to facilitate the feature extraction process. More specifically, Softmax transforms the vectors extracted from the multi-scale attentions and normalizes them into probabilities with values between [0, 1]. The higher the probability, the more attention is given to that voxel area and feature channel. This conversion into probabilities serves as an interpretation mechanism, facilitating the understanding of which features contribute more to the representation of the data. Softmax increases the distinctions between the scores by exponentiating them. This is advantageous specifically for feature extraction, since it highlights the most significant elements while reducing the importance of the less relevant ones. Additionally, an identity convolutional operation with a single feature channel is applied to summarize the information acquired from each channel extracted from Equation 5, and the sigmoid activation function is placed at the end to obtain the attention scores:

(Equation 6) $$SigmOut=Sigmoid\left(Softmax\left({FMA}_{d,w,h}*{FPR}_{d,w,h}\right)*{\;I}^{c=1}\right)\text{,}$$

where ${\;I}^{c=1}$ is the identity convolutional operation with a single channel, Softmax is the Softmax activation function, and Sigmoid is the sigmoid activation function. Equation 7 expresses the element-wise multiplication of the ${FPR}_{d,w,h}$ and the SigmOut operation:

(Equation 7) $$MSAG=SigmOut*{FPR}_{d,w,h}\text{,}$$

where MSAG is the outcome of MSAG mechanism.

### Performance evaluation

For the purpose of the study, we employed a comprehensive evaluation scheme consisting of five widely used segmentation metrics: Dice score, Hausdorff distance, average surface distance, recall, and precision. Additionally, the number of parameters for each model was reported to present models’ complexity. The selection of these metrics was motivated by specific considerations. The Dice score serves as a standard measure for assessing the overlap between the ground truth and predicted binary masks (PR), which correspond to the predictions of each trained model. Conversely, precision quantifies the fraction of predicted lesion voxels that are true positives, while recall assesses the fraction of ground-truth lesion voxels accurately identified by the model. The Hausdorff distance and the average surface distance are distance-based metrics that rely on the distances of voxels between the ground truth and PR while providing complementary insights: the former provides a worst-case scenario for each patient case, whereas the latter delivers a more robust evaluation by calculating the mean distance between the ground truth and PR for each patient case. A disadvantage of distance-based metrics is that they are not fractioned, indicating that the inability of a model to produce a mask leads to extreme outliers approximating infinity. That being said, we report the median value for the distance-based metrics (Hausdorff distance and average surface distance).
